# Automatic detection of early gastric cancer in endoscopy based on Mask region-based convolutional neural networks (Mask R-CNN)(with video)

**DOI:** 10.3389/fonc.2022.927868

**Published:** 2022-10-20

**Authors:** Jing Jin, Qianqian Zhang, Bill Dong, Tao Ma, Xuecan Mei, Xi Wang, Shaofang Song, Jie Peng, Aijiu Wu, Lanfang Dong, Derun Kong

**Affiliations:** ^1^ Key Laboratory of Digestive Diseases of Anhui Province, Department of Gastroenterology, The First Affiliated Hospital of Anhui Medical University, Hefei, China; ^2^ School of Computer Science and Technology, University of Science and Technology of China, Hefei, China; ^3^ Research and Development Department, Hefei Zhongna Medical Instrument Co. LTD, Hefei, China

**Keywords:** artificial intelligence - AI, region-based convolutional neural networks (R-CNN), endoscopy, Early gastric cancer (EGC), white light imaging, narrow band imaging (NBI)

## Abstract

The artificial intelligence (AI)-assisted endoscopic detection of early gastric cancer (EGC) has been preliminarily developed. The currently used algorithms still exhibit limitations of large calculation and low-precision expression. The present study aimed to develop an endoscopic automatic detection system in EGC based on a mask region-based convolutional neural network (Mask R-CNN) and to evaluate the performance in controlled trials. For this purpose, a total of 4,471 white light images (WLIs) and 2,662 narrow band images (NBIs) of EGC were obtained for training and testing. In total, 10 of the WLIs (videos) were obtained prospectively to examine the performance of the RCNN system. Furthermore, 400 WLIs were randomly selected for comparison between the Mask R-CNN system and doctors. The evaluation criteria included accuracy, sensitivity, specificity, positive predictive value and negative predictive value. The results revealed that there were no significant differences between the pathological diagnosis with the Mask R-CNN system in the WLI test (χ^2^ = 0.189, P=0.664; accuracy, 90.25%; sensitivity, 91.06%; specificity, 89.01%) and in the NBI test (χ^2^ = 0.063, P=0.802; accuracy, 95.12%; sensitivity, 97.59%). Among 10 WLI real-time videos, the speed of the test videos was up to 35 frames/sec, with an accuracy of 90.27%. In a controlled experiment of 400 WLIs, the sensitivity of the Mask R-CNN system was significantly higher than that of experts (χ^2^ = 7.059, P=0.000; 93.00% VS 80.20%), and the specificity was higher than that of the juniors (χ^2^ = 9.955, P=0.000, 82.67% VS 71.87%), and the overall accuracy rate was higher than that of the seniors (χ^2^ = 7.009, P=0.000, 85.25% VS 78.00%). On the whole, the present study demonstrates that the Mask R-CNN system exhibited an excellent performance status for the detection of EGC, particularly for the real-time analysis of WLIs. It may thus be effectively applied to clinical settings.

## Introduction

Gastric cancer (GC) is one of the five most common types of malignant tumors worldwide, and is regarded as the fourth cause of cancer-related mortality (1). The five-year survival rates of patients with early gastric cancer (EGC) and advanced gastric cancer (AGC) are 95% and 30%, respectively (2). Therefore, the early detection and diagnosis of gastric cancer are crucial measures to reduce the mortality rate associated with gastric cancer.

Endoscopy with white light images (WLIs) is recommended as a standard procedure for the detection of EGC (3). However, some minimal changes are easily ignored in WLIs, which often leads to the missed diagnosis of EGC. Previous studies have proven that magnifying endoscopy associated with image-enhanced endoscopy techniques can effectively improve the performance in detecting EGC (4, 5). However, this technique cannot be easily implemented in rural or undeveloped areas owing to a lack of advanced devices and experienced endoscopists.

In the face of these issues, the development of practical clinical tools is necessary. With the development of computer-assisted technology, artificial intelligence (AI) has begun to be applied to medicine, and there have been preliminary studies in the early detection of diseases, pathological diagnosis and prognosis assessment, such as liver fibrosis staging diagnosis (6), skin cancer classification (7), and diabetic retinopathy (8). Previous studies have reported the application of AI in identifying esophageal cancer (9, 10) and intestinal polyps (11), and AI has also demonstrated to be of effectively assistance in the field of endoscopic systems. At present, the object detection algorithms based on deep learning are commonly used in lesion detection in endoscopy (12, 13). These algorithms have limitations, such as large calculation and low expression accuracy (14, 15). In the early stage of the experiment, we used a small number of samples to train and validate several models, and found that the mask region-based convolutional neural network (Mask R-CNN) model was the optimal model at this stage. As a small and flexible universal object instance segmentation framework, Mask R-CNN can achieve rapid and accurate multi-object detection in the same network, while meeting the accuracy requirements of semantic segmentation. It has been found that Mask R-CNN has a good transfer learning ability (16). The present study developed an algorithm for detecting EGC based on Mask R-CNN. Prospective and controlled trials were conducted to examine the performance of the Mask R-CNN system in diagnosing EGC and to assess the clinical suitability of the Mask R-CNN system compared with endoscopists.

## Materials and methods

### Data sources

Images and videos of WLIs and narrow band images (NBIs) were collected from patients, who underwent magnifying endoscopy and narrow band imaging, as well as received endoscopic submucosal dissection as the initial treatment between October, 2017 and March, 2022. The retrospective data were obtained from previous clinical diagnosis and treatment, and the exemption of patient informed consent was received from the hospital (the First Affiliated Hospital of Anhui Medical University; Ethical no. PJ2021-08-12). All participants in the prospective trial signed informed consent forms before the trial.

An upper gastrointestinal endoscope (GIF Q260J, GIF H260Z and MAJ-290; Olympus Corporation) with magnification and NBI functions was used to observe gastric mucosal lesions. Two endoscopists with >10 years of experience used the NBIs retrospectively to identify EGC according to the vessel and surface classification (17).

All patients had biopsies or excision specimens which were histologically evaluated by a pathologist according to the revised Vienna gastrointestinal epithelial tumor classification (18), type 4 (high-grade mucosal tumors) and non-invasive muscular lesions of type 5 (invasive tumors) were defined as EGC, and the pathological result was considered as the gold standard.

Previous studies have found that EGC is difficult to distinguish from gastritis due to inflammatory cell infiltration and it often resulting in the underdiagnosis of EGC (19). In this study, the Mask R-CNN system was developed to differentiate EGC from gastric diseases such as gastritis and gastric erosion. The selection criteria were as follows: i) Patients were aged 18 to 80 years; ii) Only gastritis and gastric erosion were included in the non-cancerous images; iii) lesions comprised >30% of the entire image and could be recognized by the endoscopist. The exclusion criteria were as follows: i) Patients aged <18 or > 80 years; ii) images of polyps, ulcers or ulcer scars, remnant stomach cancer and other lesions; iii) images of mucus, blood, or other foreign bodies attached; iv) images that were difficult to evaluate due to insufficient focus, halos, bubbles or excessive blur; and v) dye-stained images.

**Figure 1:**
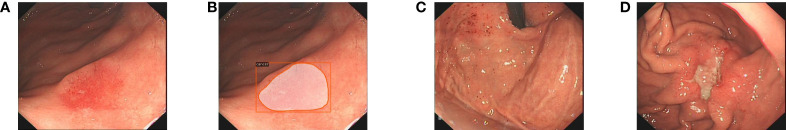
The endoscopists and the Mask R-CNN system delineate the lesion in WLI. **(A)** The EGC lesion in WLI. **(B)** The Mask R-CNN system delineates the EGC lesion. **(C)** Gastritis in WLI. **(D)** The AGC lesion in WLI. WLI, white light imaging; Mask R-CNN, mask region-based convolutional neural network; EGC, early gastric cancer; AGC, advanced gastric cancer.

**Figure 2:**
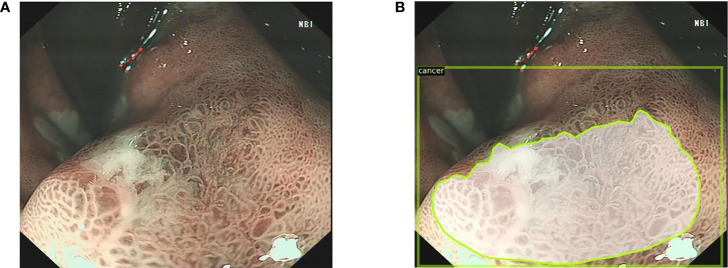
The endoscopists and the Mask R-CNN system delineate the lesion in narrow band images. **(A)** The narrow band image with the EGC lesion. **(B)** The Mask R-CNN system delineates the EGC lesion. Mask R-CNN, mask region-based convolutional neural network; EGC, early gastric cancer.

All images were marked by two endoscopists (JJ, QZ) who were trained prior to collecting the data from the electronic medical record. One endoscopist first used LabelMe (version 1.1; IBM Inc.) or Pair programming software (version 2.0; Shenzhen Duying Medical Technology Co., Ltd.) to mark the boundaries of the lesion, and the other endoscopist examined the mark; the two endoscopists then took turns in labeling work. To avoid individual bias in the image selection and labeling, delineation was confirmed only when the two endoscopists reached a consensus. Subsequently, another endoscopist (DK) who is a specialist at the First Affiliated Hospital of Anhui Medical University confirmed the images with the indicated criteria (**Figures 1**, **2**).

### Algorithm training and model recognition process

**Figure 3:**
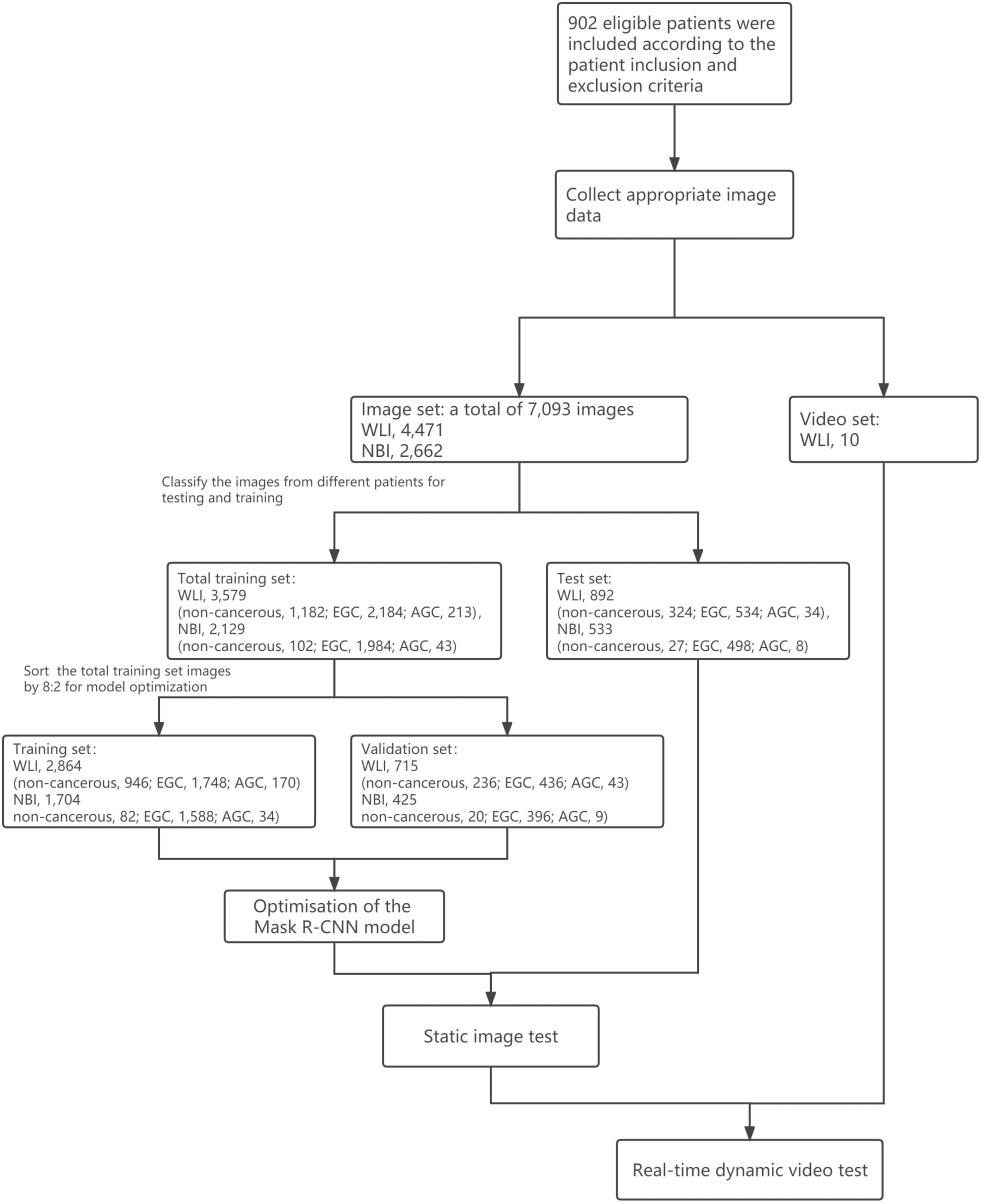
Model training and testing process.

A total of 7,093 images were retrospectively obtained from the First Affiliated Hospital of Anhui Medical University, including 4,471 WLIs and 2,662 NBIs. According to the Holdout validation method, the most common ratio for dividing the training and the validation set is 8:2. We selected 5,708 of these images to train and optimize the model based on this ratio, including 3,579 WLIs (non-cancerous, 1,182; EGC, 2,184; AGC, 213) and 2,129 NBIs (non-cancerous, 102; EGC, 1,984; AGC, 43). The 2,184 EGC images in WLI were derived from 524 EGC lesions in 522 patients while the 1,984 EGC images in NBI were obtained from 276 EGC lesions in 276 patients. The flow of the model training and test data was shown in **Figure 3**.

A Mask R-CNN object detection neural network model was used for early gastric cancer detection. By inputting multiple batches of training images and using the Back-Propagation algorithm, the network parameters are updated iteratively until the loss function converges.

**Figure 4:**
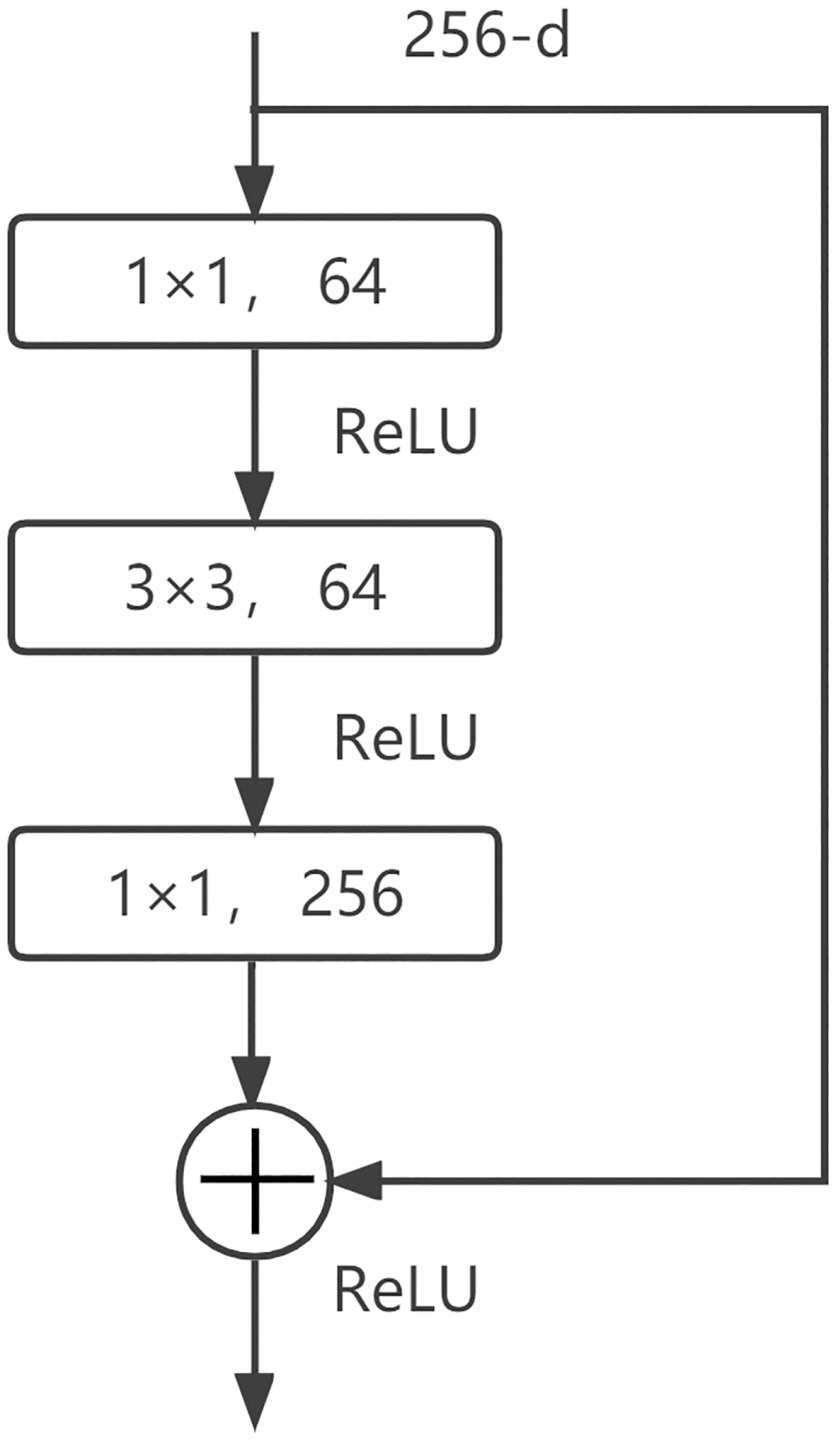
ResNet residual module. ReLU, Rectified Linear Unit.

Mask R-CNN is based on the improved implementation of object detection framework Faster-RCNN, and its architecture mainly consists of three parts. The first part is the backbone network, which is used to extract feature images from input images. The backbone structure of the model is ResNet-50, which has 50 layers of neurons. This structure first went through the convolutional layer, batch normalization layer, ReLU activation function and the calculation of the maximum pooling layer, and was then connected to four residual modules with a bottle-neck layer (**Figure 4**). Each module also has a convolution layer and ReLU activation function. The residual structure can increase the depth of the training network, learn more complex image features and eliminate the factors that may reduce the learning effect.

**Figure 5:**
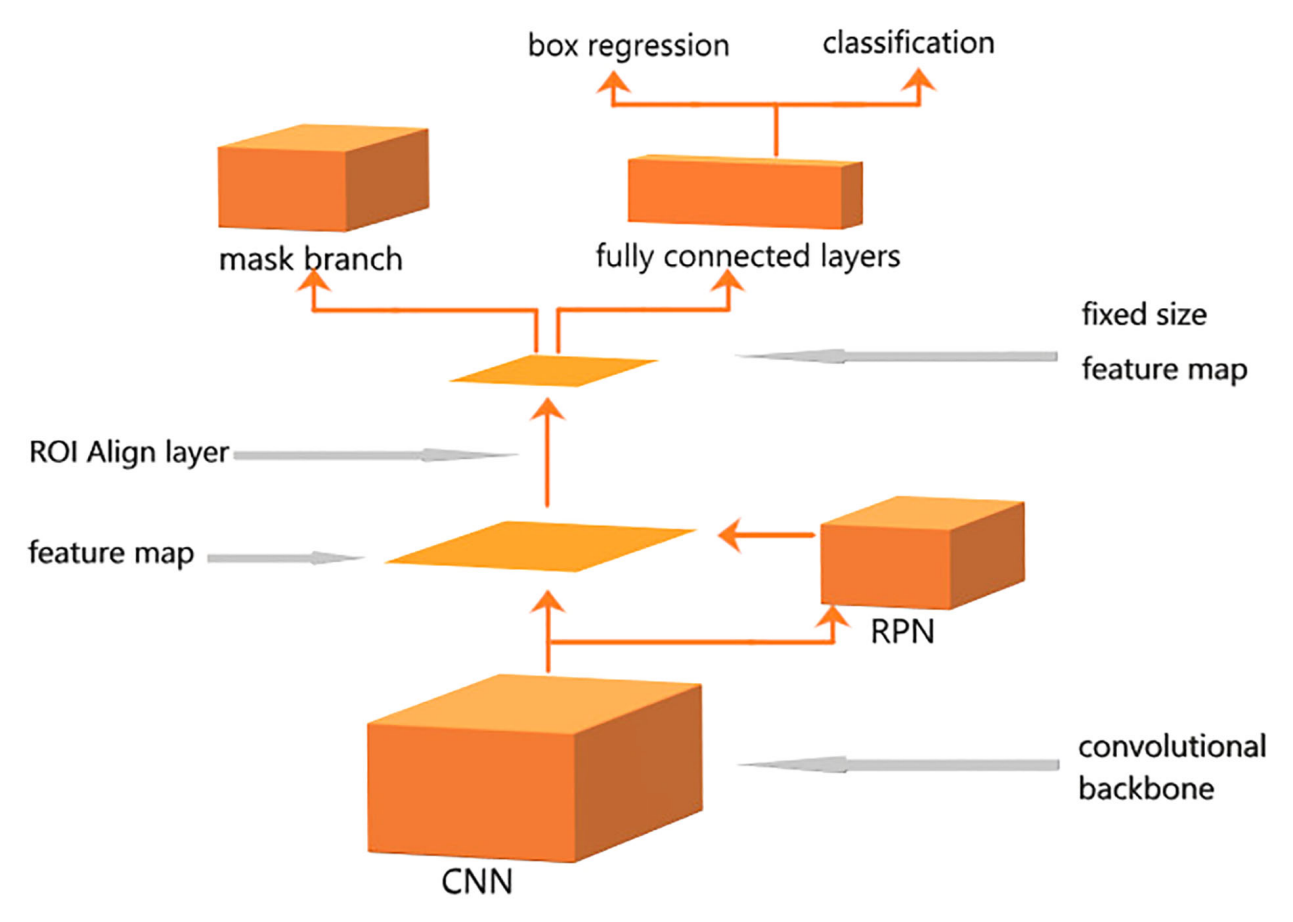
Mask region-based convolutional neural network architecture.

The pre-training weights of ImageNet were used as the initial training values of all neurons in the backbone network, as the experiment was better performed in this manner, according to the results. The backbone network will extract image features as a series of vectors to the second part of the entire architecture, ROI Align layer. The features of the image can be extracted and the proposals can be obtained. The third part is the ROI head. As the decision-making layer, in this stage, in addition to the precise prediction of the types and positions of the candidate frames, the fully convolutional network branch is added, and the image binary mask is performed to obtain pixel-level image segmentation results. The results of Mask R-CNN model output include the confidence of classification, that is, the probability of classification, and the intersection over union (IoU) of bounding boxes. The IoU is defined as follows:


$$\text{IoU }=\;\frac{{\text{area}}_{\text{pr }}\cap^{}{\text{ area}}_{\text{gt}}}{{\text{area}}_{\text{pr }}\cup^{}{\text{ area}}_{\text{gt}}}$$

where areapr is the bounding box predicted by the neural network, and areagt is the bounding box of the real label. The overall framework of Mask R-CNN is illustrated in **Figure 5**.

### Test of the Mask R-CNN system in WLIs and NBIs

The test of the Mask R-CNN system in WLIs and NBIs contained three types of lesions: Non-cancerous, EGC and AGC. In the complete image set, we prepared 892 WLIs (non-cancerous, 324; EGC, 534; AGC, 34) and 533 NBIs (non-cancerous, 27; EGC, 498; AGC, 8) as static image test sets. This EGC WLIs were from 125 patients with an average age of 64.1 years, 71.2% of whom were male. There were 63 lesions (50.4%) located in the cardia. The mean size of the EGC was 2.52 x 1.35 cm^2^ and the most common macroscopic type was type 0-II with 89 (71.2%). Sixty-five lesions (52%) were located in the muscular mucosa and 42 lesions (33.6%) in the lamina propria. The EGC NBIs were from 276 patients with an average age of 63.46 years, 74.6% of whom were male. There were 33 lesions (49.25%) located in the cardia. The mean size of the EGC was 2.33 x 1.41 cm^2^. Forty-six lesions (68.66%) were type 0-II. Thirty-three lesions (49.25%) were located in the muscular mucosa and 23 lesions (34.33%) in the lamina propria.

In total, nine different execution thresholds were hypothesized from 10 to 90% for the Mask R-CNN system and 10% was used as the interval. After the Mask R-CNN system detects the lesion, it generates a bounding box and evaluates the likelihood that the lesion is EGC. Only when the confidence of the bounding box exceeded the execution threshold, would the lesion be judged as EGC. The recognition of EGC by the Mask R-CNN system is considered correct and the capability of the Mask R-CNN system was evaluated by the recognition of EGC.

### Test of the Mask R-CNN system in white light real-time videos

In total, 10 endoscopic videos of 10 patients were prospectively obtained, and the pathological diagnosis of the biopsy or excision specimens of all patients was EGC. Of the 10 patients, 8 were male and 2 were female, with a mean age of 67.2 years. There are 7 lesions (70%) located in the cardia. This 10 lesions were all type 0-II, of which 6 were 0-IIc, and the mean size was 2.49 x 1.21 cm^2^. Nine lesions were located in the mucosal layer.

The acquired EGC videos were then edited, the WLI fragments that were not electrocoagulated were retained and the clips were reassembled into complete videos. Based on the Mask R-CNN algorithm system, the average time of the 10 videos was 270.9 sec (range, 105 to 422 sec). Video streaming was up to 35 frames/sec.

After converting the video into several images according to the corresponding ratio, the endoscopist screened out the images containing the EGC lesions and classified them. The Mask R-CNN system was then used to identify all images. In the video test, the Mask R-CNN system added a voting screening module, that is, voting on the prediction processing results of video content within a certain period of time, and adjusting the final output content and identification within the time period. This module can effectively reduce false positives during dynamic detection. It was stipulated that as long as >1/5 of the video frame detected the EGC target within half a second, the target can be considered to be correctly identified during this time. When there were multiple lesions on a picture at the same time, the highest priority was to identify EGC, and only positive results were identified as EGC.

### Test of the Mask R-CNN system vs. Endoscopists

In this controlled experiment, normal mucosal images were added and AGC images were subtracted to achieve the purpose of detecting EGC in different mucosal environments. A total of 743 images of normal mucosa and 858 images containing EGC, non-cancerous in WLIs were prepared, from which a total of 400 images were randomly selected according to the ratio of normal:non-cancerous:EGC of 2:1:1 (non-EGC images:EGC images, 3:1). A total of 15 endoscopists from the First Affiliated Hospital of Anhui Medical University were invited to this test. They were divided into experts (endoscopic experience, >10 years), seniors (endoscopic experience, >5 years) and juniors (endoscopic experience, >1 years), and each doctor completed the test independently. The Mask R-CNN system was then used to identify the images, and the performance of the Mask R-CNN system and that of endoscopists was compared.

### Statistical analysis

The main evaluation indicators included accuracy, sensitivity, specificity, positive predictive value (PPV) and negative predictive value (NPV) (**Table 1**). The area under the receiver operating characteristic (ROC) curve (AUC) was calculated to obtain the optimal threshold and evaluate the performance of the Mask R-CNN system in the image test and video test. The comparison between the Mask R-CNN, pathological diagnosis and endoscopists was conducted using a Chi-squared test. A value of P<0.05 was considered to indicate a statistically significant difference. Statistical analysis was performed using SPSS software (version 26.0; IBM Inc.).

**Table 1:** Evaluation criteria.

Accuracy	Correct identification number/All images
Sensitivity	Correctly identify EGC/real EGC The higher the sensitivity, the less missed the diagnosis
Specificity	Correctly identify non-EGC/real non-EGC The higher the specificity, the less misjudgments there are
Positive Predictive Value	Real EGC/identify EGC The higher the positive predictive value, the higher the true diagnosis rate identified as EGC
Negative Predictive Value	Real non-EGC/identify non- EGC The higher the negative predictive value, the higher the true non-prevalence rate identified as non-EGC

EGC, early gastric cancer.

## Results

### Performance of the Mask R-CNN system in WLIs and NBIs

**Figure 6:**
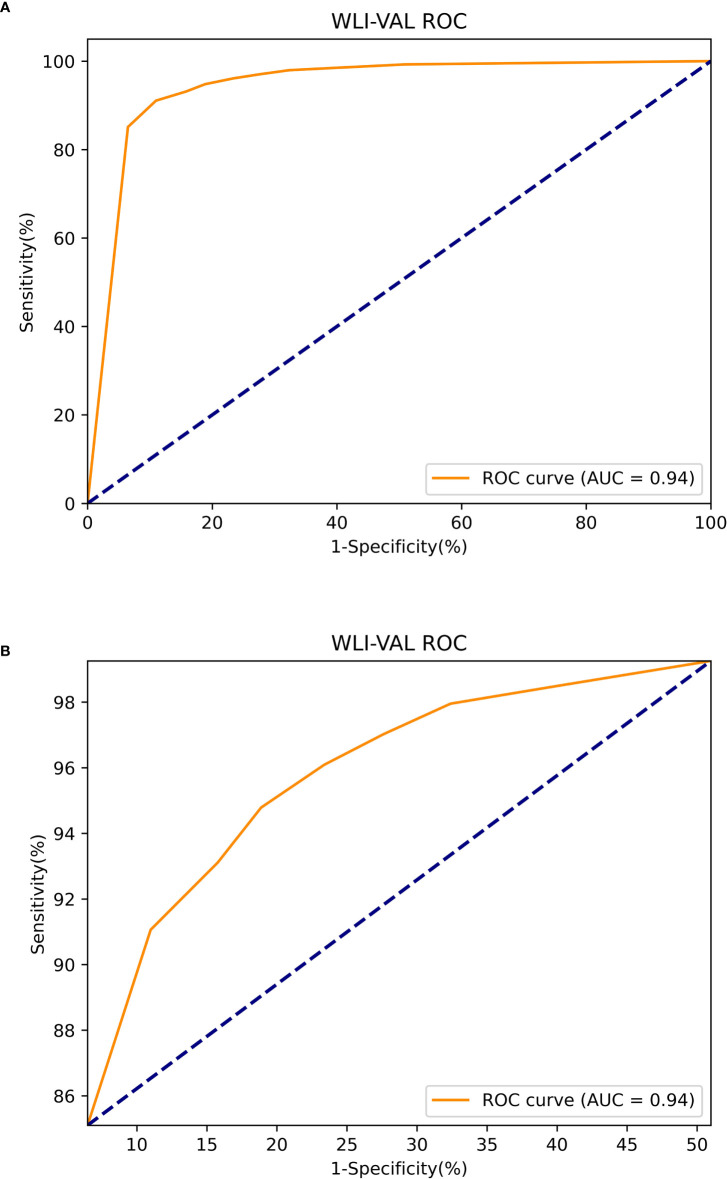
The ROC curve of the white light images test. **(A)** The global ROC curve of the white light images test. **(B)** The regional ROC curve of the white light images test.

**Figure 7:**
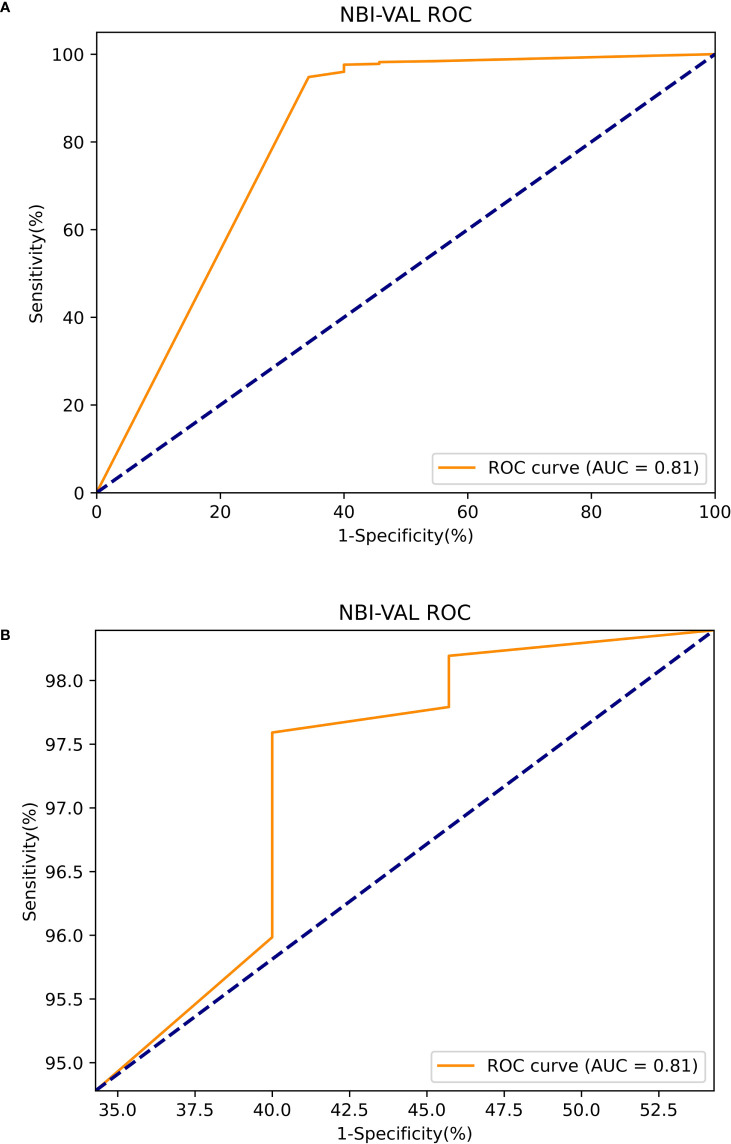
The ROC curve of the narrow band images test. **(A)** The global ROC curve of the narrow band images test. **(B)** The regional ROC curve of the narrow band images test.

The Mask R-CNN system was tested at various thresholds to obtain the ROC curve. In WLIs, the Mask R-CNN system identified EGC most effectively at a threshold of 80%, with an accuracy of 90.25%, a sensitivity of 91.06%, a specificity of 89.01%, PPV of 92.61%, NPV of 86.81% and an AUC of 0.94 (**Figure 6**). At this time, the accuracy, sensitivity and specificity of identifying non-cancerous were 83.86%, 88.50%, and 81.01%. The accuracy, sensitivity, and specificity of identifying AGC were 98.32%, 82.35%, and 98.95%. The accuracy assessment of the outline boundary (mask) is calculated using the cross-merger ratio (IoU) of the detection boundary and the true label boundary, based on the benchmark when the IoU > 0.5 is counted as the correct judgment, and the comprehensive accuracy of the outline boundary (mask) is 61.04%. Following a comparison with the diagnosis results of the pathological analysis, no notable statistically significant differences were found between the Mask R-CNN system and the pathological analysis (χ^2 =^ 0.189, P=0.664) (**Table 2**). In NBIs, the Mask R-CNN system exhibited an accuracy of 95.12%, a sensitivity of 97.59%, a specificity of 89.01%, PPV of 97.20%, NPV of 63.64%, and an AUC of 0.81 at the 50% threshold (**Figure 7**). No significant differences were found between the Mask R-CNN system and the pathological analysis (χ^2 =^ 0.063, P=0.802) (**Table 3**).

**Table 2:** Performance of the Mask R-CNN system in white light images.

Mask R-CNN	Pathology		
	EGC	Non-EGC (non-cancerous, AGC)	Total
EGC	489	39	528
Non-EGC (non-cancerous, AGC)	48	316	364
Total	537	355	892

According to the Chi-square test results, P=0.664 (>0.05). Mask R-CNN, mask region-based convolutional neural network; EGC, early gastric cancer; AGC, advanced gastric cancer.

**Table 3:** Performance of the Mask R-CNN system in narrow band images.

Mask R-CNN	Pathology		
	EGC	Non-EGC (non-cancerous, AGC)	Total
EGC	486	14	500
Non-EGC (non-cancerous, AGC)	12	21	33
Total	498	35	533

According to the Chi-square test results, P=0.802 (>0.05). Mask R-CNN, mask region-based convolutional neural network; EGC, early gastric cancer; AGC, advanced gastric cancer. Gastric cancer is one of the five most common types of malignant tumors worldwide, and is regarded as the fourth cause of cancer-related mortality. The five-year survival rates of patients with early gastric cancer and advanced gastric cancer are 95% and 30%, respectively. Therefore, the early detection and diagnosis of gastric cancer are crucial measures to reduce the mortality rate associated with gastric cancer. Endoscopy with white light images is recommended as a standard procedure for the detection of early gastric cancer. However, some minimal changes are easily ignored in WLIs, which often leads to the missed diagnosis of EGC. With the development of computer-assisted technology, artificial intelligence has begun to be applied to solve this problem. Existing study often focus on only individual aspects, such as high accuracy or high precision.This study used the Mask R-CNN algorithm, tested separately under WLI and NBI, and compared with endoscopists. This AI model can achieve high accuracy in identifying EGC and depict the boundaries of EGC lesions. At the same time, the AI model can meet the high-speed recognition requirements of gastroscopy in clinical environments.

### Performance of the Mask R-CNN system in white light real-time videos

The Mask R-CNN system had the same detection capabilities of testing both local videos and real-time videos. The Mask R-CNN system can test local videos without frame limits. It also can capture real-time videos up to 35 frames/s and output results with a delay of 80 msec, enabling most clinical environments (**Video S1**).

**Figure 8:**
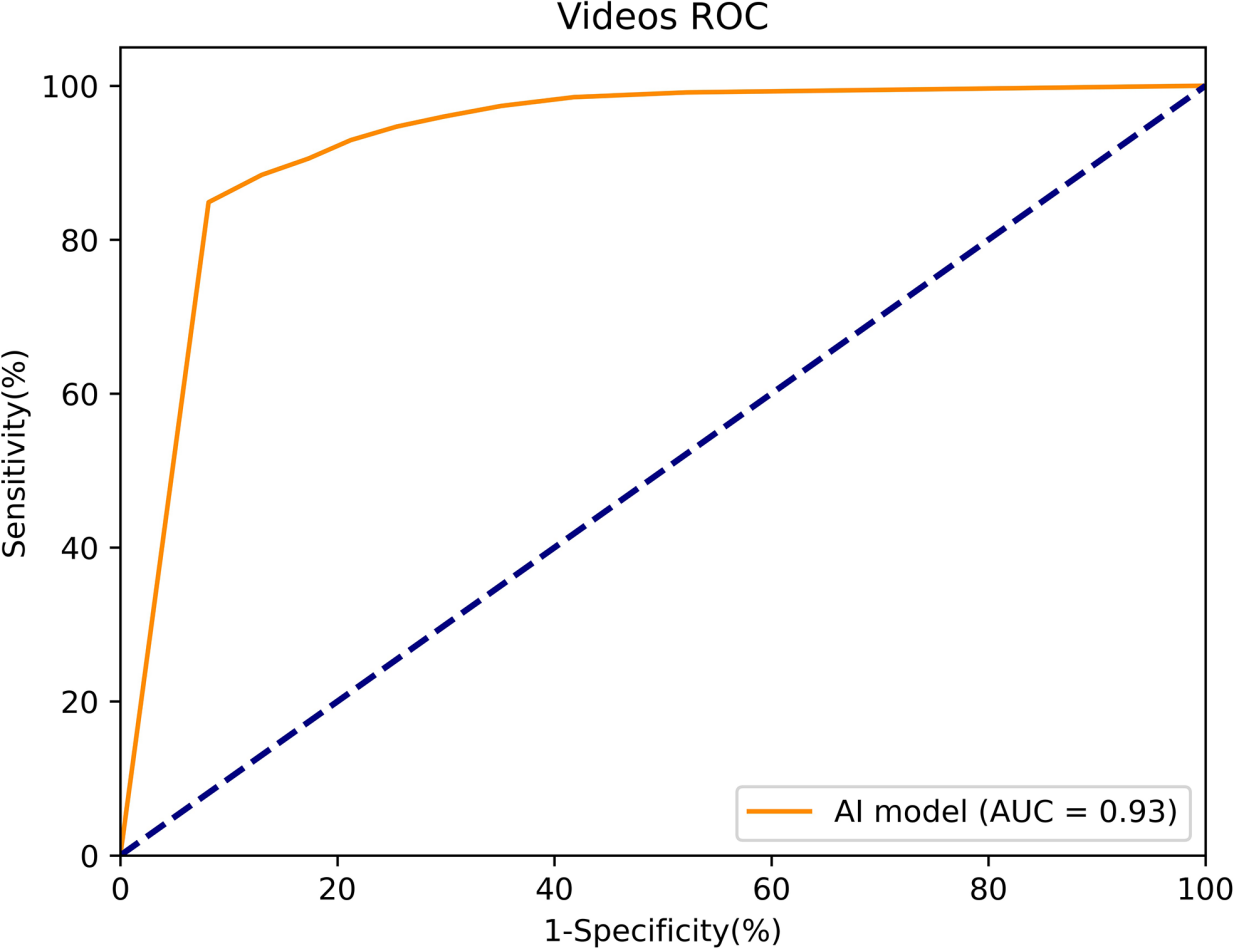
The ROC curve of the white light real-time videos test.

When the threshold was 90%, the Mask R-CNN system obtained better results on the video stream. The accuracy, sensitivity and specificity of diagnostic EGC were 90.27, 84.86 and 91.87%, respectively, and the PPV and NPV were 75.47 and 95.37%, respectively. The AUC of the RCNN system was 0.93 (**Figure 8**).

### Performance of the Mask R-CNN system vs. Endoscopists

**Figure 9:**
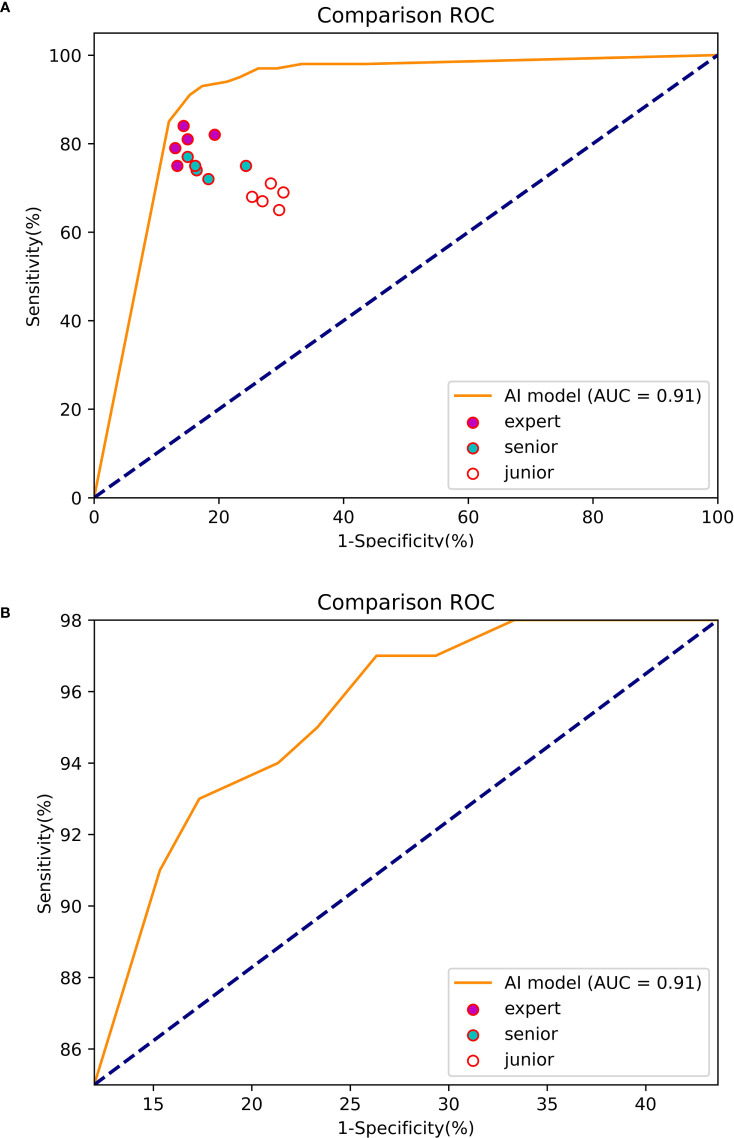
The ROC curve of the experiment between the Mask R-CNN system and Endoscopists. **(A)** The global ROC curve of the controlled experiment. **(B)** The regional ROC curve of the controlled experiment.

At the 70% threshold, the various indicators of the Mask R-CNN system were relatively balanced, with an accuracy, sensitivity, and specificity of 85.25, 93 and 82.67%, respectively, and the PPV and NPV were 64.14 and 97.25%. The AUC was 0.91 (**Figure 9**).

The accuracy (83.80%), sensitivity (80.20%) and specificity (85.00%) of the experts in identifying EGC were significantly higher than those of seniors (accuracy of 78.00%, sensitivity of 74.60% and specificity of 79.13%) and juniors (accuracy of 70.90%, sensitivity of 68.00% and specificity of 71.87%).

The sensitivity of the Mask R-CNN system was 93.00%, which was significantly higher than that of the experts and the difference was statistically significant (χ^2 =^ 7.059, P=0.000). The specificity was higher than that of the juniors (χ^2 =^ 9.955, P=0.000) and the overall accuracy rate was higher than that of the seniors (χ^2 =^ 7.009, P=0.000) (**Table 4**).

**Table 4:** Comparison between the Mask R-CNN system and endoscopists.

Diagnoser	Accuracy	Sensitivity	Specificity
Mask R-CNN % (n/total)	85.25 (341/400)	93.00 (93/100)	82.67 (248/300)
Experts % (95% CI)	83.80 (81.70, 85.90)	80.20 (75.95, 84.45)^a^	85.00 (81.84, 88.17)
Seniors % (95% CI)	78.00 (75.88, 80.12)^a^	74.60 (72.34, 76.86)^a^	79.14 (76.07, 82.15)
Juniors % (95% CI)	70.90 (68.87, 72.93)^a^	68.00 (65.22, 70.78)^a^	71.87 (69.36, 74.38)^a^

a Compared to artificial intelligence, P<0.05. Mask R-CNN, mask region-based convolutional neural network.

## Discussion

Gastric cancer is the most common type of cancer and it is the fourth leading cause of cancer-related mortality worldwide (1). Considering the notable differences between EGC and AGC, early detection and diagnosis are crucial for patients. An endoscopy is recognized as the standard method for detecting EGC, and endoscopic pathology is considered the gold standard for diagnosing EGC (3, 20). However, it is difficult to distinguish the miniscule changes of the mucosa in traditional WLIs. The successful detection of lesions mainly depends on the professional skills and experience of the endoscopist (21). Studies have validated the effectiveness of WLIs in diagnosing EGC, although the sensitivity is low. In recent years, several more advanced techniques, such as magnifying endoscope, NBI, automatic fluorescence imaging and blue laser imaging have been applied to EGC detection (4, 5), and studies have demonstrated that a magnifying endoscope combined with NBI technology for the diagnosis of EGC has a better sensitivity and specificity than conventional WLI (22). However, the small field of view of the magnification endoscope and the insufficient brightness of the NBI are not suitable for routine screening. In addition, the use of these advanced technologies has certain requirements for endoscopic equipment and endoscopists, and this remains a challenge for hospitals in underdeveloped areas (23).

AI is based on the computing and learning ability of machines, which can efficiently solve problems (24). It was widely used and developed in the field of medical image recognition, such as radiological diagnosis (6), skin cancer classification (7), diabetic retinopathy (8) and histopathology (25).

The combination of AI and endoscopic systems is mainly manifested in gastrointestinal tumor screening and predicting the depth of invasion (9, 26). Ikenoyama Y et al. (27) developed a CNN system that specifically identifies early cancer lesions in WLI still images. The CNN system showed a higher diagnostic sensitivity (58.4% vs 31.9%) and faster diagnosis in an experiment comparing the diagnostic ability of 67 endoscopists. On the downside, the system uses rectangular boxes to identify lesions and does not allow dynamic identification of early cancers, and its diagnostic capability in still images could be improved. Nam et al. (28) designed three consecutive submodels to identify single stationary images. The heatmap display generated by gradient-weighted class activation mapping (Grad-CAM) was used for comparison. This model indicates the rough edges of lesions; however, the use of heatmap display has a greater impact on the endoscopists’ observation of the mucosa. Tang et al. (29) developed a real-time AI assist system for detecting EGC that can diagnose at a rate of 15 msec per image, sufficient to handle gastroduodenoscopic video streams. In the 26 videos of the experiment, 23 lesions were detected and the sensitivity was 88.5%. Wu et al. (30) developed an AI system termed ENDOANGEL-LD, trained with multicenter, large sample data to detect gastric mucosal lesions and gastric tumors from many different types of gastric lesions. The study performed well in both WLI still images and videos. Particularly in the experiment of diagnosing gastric tumors by videos, the accuracy of ENDOANGEL-LD was 72.0% (72/100) and the sensitivity was 100.0% (38/38), which was higher than the four experts (accuracy, 68.0%; sensitivity, 85.5%). The study also designed prospective trials to achieve high sensitivity and specificity in successive patients, initially demonstrating the potential of ENDOANGEL-LD to assist endoscopists in their clinical work. However, using the number of videos as a unit of judgment is not sufficient to illustrate the ability of ENDOANGEL-LD in dynamic detection. Wu et al. (31) proposed a randomized controlled trial to evaluate the performance of AI systems for catching blind spots during endoscopy and detecting EGC in real-time. The mean [standard deviation (SD)] for the total time of the system outputs predicted was 230 (SD 60) msec. Therefore, it was set to process videos in real-time at two frames per second. The study converted videos into images at the corresponding frame rate for the first time; however, the performance of AI in this case has not yet been evaluated. Hu et al. (32) used deep learning to recognize EGC under ME-NBI images with an accuracy rate of 77.0% and a sensitivity of 79.2%, which can be similar to that of experts. This study used Grad-CAM to visualize the area where the model predicts the most likely. Ueyama H et al. (33) also designed an AI model to identify EGC under ME-NBI images, which used the more accurate Heatmap of occlusion analysis marker and obtained high sensitivity (98%) and specificity (100%). However, these models only worked with ME-NBI images, but not with videos. This requires the endoscopist to first suspect EGC under the WLI prior to initiating the next procedure.

In recent years, with the development of computer-aided technology, a variety of deep learning models have been applied to endoscopic systems. In terms of the lesion detection of endoscopic images, deep learning-based object detection algorithms are usually used, which are mainly divided into two categories. The first category is the one-stage object detection algorithm represented by ‘you only look once’, which has a high detection speed; however, the detection accuracy still needs to be improved (34). The other type is the two-stage algorithm represented by the dynamic convolutional neural network (DCNN), Fast RCNN, Faster RCNN, etc., based on CNN and regional candidate networks, which has a higher detection accuracy than the one-stage algorithm (35, 36). The learning ability of DCNN has been greatly improved; however, the training time is too long and the practicality is poor; Fast RCNN requires the time-consuming extraction of candidate regions for images; the efficiency of Faster RCNN has improved, although the requirements for experimental equipment are high and the training time is lengthy. At the same time, Faster RCNN only obtains a rough representation (35, 36).

In view of the existing gastric cancer screening studies, such as a large training sample size, long training time, large calculation, slow running speed and low expression accuracy, a Mask R-CNN based AI system was developed as a novel technology to assist in the endoscopic diagnosis of EGC. Mask R-CNN integrates the previous excellent deep learning research results; it can not only perform multi-object detection rapidly and accurately in the same network, but can also complete semantic segmentation and achieve high accuracy target outlines at the pixel level. Mask R-CNN also delegates the four steps of candidate region generation, feature extraction, classifier classification and regressor regression to the deep neural network, which is not only relatively small in size, but also faster in calculation. It has been demonstrated that the algorithm has a good transfer learning ability, and it can achieve a better learning effect with a lower amount of data training (16).

The Mask R-CNN system was trained on an appropriate sample size and performed well in WLIs with an accuracy of 90.25%, a sensitivity of 91.06%, a specificity of 89.01%, a PPV of 92.61% and a NPV of 86.81%. It also exhibited excellent diagnostic performance (accuracy, 95.12%; sensitivity, 97.59%; PPV, 97.20%) in the NBI test. There were no significant differences between the AI and the pathological diagnosis results. In the prospective video experiments, the Mask R-CNN system was able to identify EGC lesions (100%) in all videos. Unlike previous studies (29–31), in order to further evaluate its performance, the video streams were converted into images of the corresponding frame number for detection. While maintaining a high performance (90.27, 84.86 and 91.87% accuracy, sensitivity and specificity for the diagnosis of EGC, respectively), the latency of acquiring data and outputting results is only 80 msec, and it can process videos at 35 frames per second. In the controlled trial, the overall performance of the Mask R-CNN system was no worse than that of the experts, providing a basis for AI-assisted endoscopic diagnosis. In addition, the present study demonstrated that the AI model identified independent EGC in multi-sample test images consisting of normal mucosa, inflammation non-cancerous, EGC and AGC.

The system was tested in both WLI and NBI data. When it recognized EGC under WLI, it can further switch to the NBI recognition mode. This design simulates as much as possible the actual operation process of endoscopists in clinical examination and provides a basis for the future application of the Mask R-CNN system to the clinical environment.

It is worth noting that, in the image display of the existing research, the majority of the lesions were illustrated in boxes or heatmaps, which were lacking in accurate range and visual field display. Similar to the previous study (37), the Mask R-CNN system in this study used images labeled by artificial polygons as training data. As a pixel-level object detection method, Mask R-CNN can accurately draw the predicted EGC lesion boundary when identifying images. It more effectively indicates the lesion range, and prompts for the next biopsy or endoscopic mucosal dissection. Accurate lesion display promises to provide new clues for EGC feature learning in the future. Compared to the algorithms used in previous studies, Mask R-CNN had better results in some indicators. At the same time, the system added a module for voting and screening of video detection results to the video processing, which effectively reduced the false alarm rate of background processing and analysis and reduced misjudgments.

In clinical practice, the consequences of the missed diagnosis of gastric cancer are much more severe than misdiagnosis. The authors focused on the balance of sensitivity and specificity in the results of the Mask R-CNN system, and strive to achieve the most suitable results for clinically applications, minimizing EGC misses, while avoiding misjudgments as much as possible.

The present study is not without limitations however, and these should be stated. First of all, as the data were single-center, the sample size was small and the selection bias cannot be excluded. Second, the training data were all derived from manual labeling, and it may be influenced by the level of expertise of the endoscopists. In addition, no biopsy or long-term follow-up was performed for non-cancerous images, and there was a possibility of false negatives. Fourth, this study only included gastritis, gastric erosion and AGC images as the identification of EGC, and did not add images of ulcers and intestinalization, etc. At the same time, the Mask R-CNN system was only generally designed for the diagnosis of EGC, and could not better distinguish the specific types of mucosal lesions. In the future, the group will further add a variety of lesion images that are difficult to identify with EGC to improve the accuracy of the Mask R-CNN system. As image-enhanced endoscopy is rarely used in clinical examinations unless there are suspicious findings in the WLI. This study focused on the experiment of WLIs for Mask R-CNN systems and endoscopists and did not add controlled tests for NBIs. At last, the present study conducted training and testing on the existing data, and did not add test images to the training set for re-learning. This suggests that the Mask R-CNN system is lacking in self-directed learning.

In conclusion, the Mask R-CNN system developed proved its excellent performance in detecting EGC through prospective trials and controlled trials. It is expected to play a role in the training of endoscopists in underdeveloped regions and in the diagnosis of EGC in the clinical environment.

## Data availability statement

The datasets presented in this article are not readily available because patient privacy involved. Requests to access the datasets should be directed to jinjingahmu@126.com.

## Ethics statement

The studies involving human participants were reviewed and approved by Clinical Medical Research Ethics Committee of the First Affiliated Hospital of Anhui Medical University. The patients/participants provided their written informed consent to participate in this study.

## Author contributions

JJ and DK conceived and designed the study. JJ, QZ, XW, XM, SS, JP and AW contributed to the acquisition of data. JJ, QZ, BD, TM and LD contributed to the analysis and interpretation of data. JJ, QZ and DK drafted and revised the manuscript. DK supported the project. JJ and QZ confirm the authenticity of all the raw data. All authors have read and approved the final manuscript.

## Funding

The present study was supported by the research fund project of 2022 Key Research and Development Plan Project of the Science and Technology Department of Anhui Province (No. 9021240206).

## Conflict of interest

Authors SS, JP, and AW were employed by Hefei Zhongna Medical Instrument Co. LTD.

The remaining authors declare that the research was conducted in the absence of any commercial or financial relationships that could be construed as a potential conflict of interest.

## Publisher’s note

All claims expressed in this article are solely those of the authors and do not necessarily represent those of their affiliated organizations, or those of the publisher, the editors and the reviewers. Any product that may be evaluated in this article, or claim that may be made by its manufacturer, is not guaranteed or endorsed by the publisher.

## Supplementary material

Supplementary Video 1 Performance of the mask region-based convolutional neural network architecture in the real-time white light video.
