# Supplementary material for: Automatic detection of early gastric cancer in endoscopy based on Mask region-based convolutional neural networks (Mask R-CNN)(with video)
Source: Front Oncol. 2022 Oct 20;12:927868. doi: 10.3389/fonc.2022.927868 (PMC9630732; doi:10.3389/fonc.2022.927868)
Supplement: Supplementary Video 1 — Performance of the mask region-based convolutional neural network architecture in the real-time white light video. [file DataSheet_1.zip › Updated Data sheet 1(Video)/Video Caption.doc]

**Video S1.** Performance of the mask region-based convolutional neural network architecture in the real-time white light video.
